# Invasive *Streptococcus oralis* Expressing Serotype 3 Pneumococcal Capsule, Japan

**DOI:** 10.3201/eid2808.212176

**Published:** 2022-08

**Authors:** Bin Chang, Masatomo Morita, Akiyoshi Nariai, Kei Kasahara, Akira Kakutani, Michinaga Ogawa, Makoto Ohnishi, Kazunori Oishi

**Affiliations:** National Institute of Infectious Diseases, Tokyo, Japan (B. Chang, M. Morita, M. Ogawa, M. Ohnishi);; Matsue Red Cross Hospital, Shimane, Japan (A. Nariai);; Nara Medical University, Nara, Japan (K. Kasahara); Nara City Hospital, Nara (A. Kakutani);; Toyama Institute of Health, Toyama, Japan (K. Oishi)

**Keywords:** Streptococci, bacteria, *Streptococcus oralis*, serotype 3 pneumococcal capsule, mucoid phenotype, multifragment recombination, *hyl*, antimicrobial resistance, Japan

## Abstract

We report 2 adult cases of invasive disease in Japan caused by *Streptococcus oralis* that expressed the serotype 3 pneumococcal capsule and formed mucoid colonies. Whole-genome sequencing revealed that the identical serotype 3 pneumococcal capsule locus and *hyl* fragment were recombined into the genomes of 2 distinct *S. oralis* strains.

*Streptococcus oralis* is a viridans streptococcus that is divided into 3 subspecies *S. oralis* subsp. *oralis*, *dentisani*, and *tigurinus* (*1*). Differentiation between these subspecies and other α-hemolytic streptococci, including *S. pneumoniae*, remains difficult because they share similar biochemical properties. *S. oralis* inhabits the oral cavity and can cause severe infections in persons with immunodeficiency (*2*). Antimicrobial drug resistance and capsule expression studies have demonstrated that gene transfer can occur from oral *Streptococcus* spp. to *S. pneumoniae* (*3*–*5*). Most oral *Streptococcus* spp. have a pneumococcus-like capsule locus and produce capsular polysaccharides (*6*).

We report 2 cases of invasive streptococcal disease in older adults in Japan (Table). Case 1 occurred in a 69-year-old man with gastric cancer; case 2 occurred in a 78-year-old man with bacteremic meningitis who had no underlying disease. Both patients were successfully treated with antimicrobial agents. The bacterial isolates (ASP0312-Sp from case 1 and SP2752 from case 2) contained α-hemolytic bacteria that formed characteristic mucoid colonies on blood agar (Table). Quellung reactions were strongly positive for pool R or pneumococcal serotype 3 antisera (Statens Serum Institut, https://en.ssi.dk), suggesting that the isolates were *S. pneumoniae* serotype 3. However, both isolates were optochin-resistant and bile-insoluble. Moreover, multilocus sequence typing (MLST) showed that the sequences of all 7 alleles of ASP0312-Sp and 5 alleles of SP2752 differed from those registered in the MLST database (https://pubmlst.org) (Table). For SP2752, the allele numbers were 341 for *gdh* and 406 for *spi*. Furthermore, we observed nucleotide differences between ASP0312-Sp and SP2752 in *aroE* (31 different bp), *gdh* (34 bp), *gki* (25 bp), *recP* (25 bp), *spi* (14 bp), *xpt* (47 bp), and *ddl* (15 bp), which indicated that the strains were distinct. These results suggested that the 2 strains were nonpneumococcal *Streptococcus* spp.

**Table:** Characteristics of invasive *Streptococcus* *oralis* expressing serotype 3 pneumococcal capsule from 2 adult patients, Japan*

Case	Onset date	Isolate ID	Source	Positive Quellung reaction	No. different bases						
					*aroE*	*gdh*	*gki*	*recP*	*spi*	*xpt*	*ddl*
1	January 2015	ASP0312-Sp	Blood	Pool R, serotype 3	61	30	44	32	4	41	37
2	April 2014	SP2752	Blood, CSF	Pool R, serotype 3	54	–†	40	33	–†	47	36

*Each indicated allele from *S. oralis* isolates ASP0312-Sp and SP2752 was compared with corresponding alleles from *S. pneumoniae* that have been registered in the MLST database (https://pubmlst.org). The number of different bases represents the number of polymorphisms between the isolates and the *S. pneumoniae* allele with the greatest homology. Pool R and pneumococcal serotype 3 antisera (Statens Serum Institut, https://en.ssi.dk). CSF, cerebrospinal fluid; ID, identification; MLST, multilocus sequence typing; –, no different bases found. †The allele numbers were 341 for *gdh* and 406 for *spi*.

For species identification, we performed phylogenetic analyses of whole-genome sequences (Appendix). Homologous core gene clustering showed that ASP0312-Sp and SP2752 belonged to the *S. oralis* clade (Figure); they were distant from one another, which was consistent with the MLST results.

**Figure:**
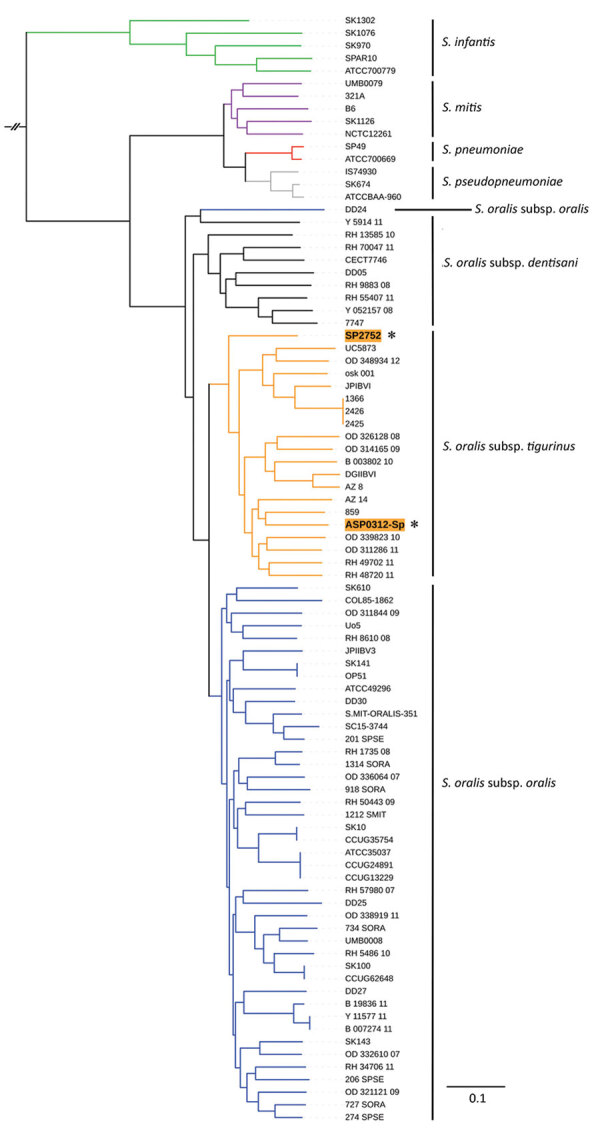
Phylogenetic analysis of invasive *Streptococcus* *oralis* expressing serotype 3 pneumococcal capsule from 2 adult patients, Japan. Asterisks and orange shading indicate genomes from isolates ASP0312-Sp and SP2752 identified in this study. Homologous core gene clusters of 71 strains from 3 *Streptococcus oralis* subsp., 2 *S. pneumoniae*, 5 *S. mitis,* 5 *S. infantis,* and 3 *S. pseudopneumoniae* were downloaded from the National Center for Biotechnology Information database (https://www.ncbi.nlm.nih.gov) and compared with the ASP0312-Sp and SP2752 genomes. Branch lengths represent the genetic distance. Scale bar indicates nucleotide substitutions per site.

To investigate recombination events, we compared the sequences surrounding the capsule loci of ASP0312-Sp and SP2752 with those of *S. oralis* subsp. *tigurinus* osk_001 and *S. pneumoniae* serotype 3 OXC141 (Appendix Figure). For ASP0312-Sp, the sequence corresponding to the downstream region of *nsik* up to the 5′ terminus of the gene encoding the cell wall binding repeat protein in osk_001 was replaced by a fragment of ≈30 kb from pneumococcus. For SP2752, the sequence encoding an ATPase up to the 5′ terminus of the gene encoding the cell wall binding repeat protein in osk_001 was replaced by a fragment of ≈16 kb from pneumococcus. The capsule sequences of ASP0312-Sp and SP2752 were 100% identical to the corresponding sequences located from 303730 to 312820 bp in HU-OH (GenBank accession no. AP018937.1), a serotype 3 pneumococcal strain that was isolated in Japan (*7*).

We performed homology searches of 36 known pneumococcal virulence genes because multifragment recombination has been demonstrated during the capsular transformation process in pneumococcal populations (*8*). In ASP0312-Sp and SP2752, the *hyl* gene, which encodes hyaluronate lyase (*9*), was located distantly from the capsule locus and shared 96% identity with that of *S. pneumoniae.* We did not detect homologs of the other 35 genes for either isolate. 

A recent study reported that acapsular pneumococcus became virulent after transformation with the capsule gene from SK95, which is an oral *S. mitis* strain (*5*). This previous study demonstrated a cross-species transformation from a commensal streptococcal species to pneumococcus (*5*). Our results complement this report, although the direction of transformation in our study was reversed. Our analyses of 2 human patients with invasive disease caused by *S. oralis* provided evidence of cross-species gene transfer from pneumococcus to a commensal streptococcal species. Acquisition of capsule and *hyl* genes might have increased pathogenicity (*9*,*10*) and contributed to progression of invasive disease in these 2 cases.

In conclusion, because of discrepancies between phenotypic and biochemical analyses, we used MLST and whole-genome sequencing to identify streptococcal species in these 2 patients. Our study indicates a potential pitfall for identifying and serotyping pneumococci that can occur if the bacteria are not isolated. Thus, when α-hemolytic streptococci are isolated from a sterile site, clinicians should request molecular analyses to identify the causative species, regardless of the mucoid phenotype.

Appendix Additional information for invasive *Streptococcus oralis* expressing serotype 3 pneumococcal capsule, Japan.
