# Supplementary material for: Invasive Streptococcus oralis Expressing Serotype 3 Pneumococcal Capsule, Japan
Source: Emerg Infect Dis. 2022 Aug;28(8):1720–2. doi: 10.3201/eid2808.212176 (PMC9328907; doi:10.3201/eid2808.212176)
Supplement: Appendix — Additional information for invasive Streptococcus oralis expressing serotype 3 pneumococcal capsule, Japan. [file 21-2176-Techapp-s1.pdf]

Article DOI: https://doi.org/10.3201/eid2808.212176

Invasive *Streptococcus oralis* Expressing Serotype 3 Pneumococcal Capsule, Japan

Appendix

Culturing Methods, Biochemical Tests, Serotyping, and Multilocus Sequence Typing (MLST)

The isolated streptococcal strains were incubated at 37°C on agar plates prepared with BD Columbia Agar with 5% Sheep Blood (Becton Dickinson, https://www.bd.com). Optochin susceptibility and bile solubility tests were performed as described elsewhere (*1*). Quellung reaction tests with pneumococcal antisera (Statens Serum Institut, Copenhagen, Denmark) were performed in accordance with the manufacturer's protocol. MLST was performed according to the method for *S. pneumoniae* described on the MLST website (https://pubmlst.org/spneumoniae). Sequences of the *aroE*, *gdh*, *gki*, *recP*, *spi*, *xpt*, and *ddl* alleles were assembled and compared with the registered sequences listed on the website.

Whole-Genome Sequencing and Phylogenetic Analysis

After single-colony isolation, genomic DNA from ASP0312-Sp and SP2752 was purified by using the DNeasy Blood & Tissue Kit (QIAGEN, https://www.qiagen.com). Genomic DNA

libraries were constructed by using the Nextera XT DNA Library Preparation Kit (Illumina, San Diego, CA, USA) and then sequenced by using a MiSeq system (Illumina). Genome assembly was performed by using SPAdes version 3.13.1 with the --careful option and a read coverage cutoff value of 10 (*2*). For species identification, a phylogenetic analysis of the whole-genome sequence was performed. The sequences of 86 streptococcal strains (*Streptococcus oralis* subsp. *oralis*, n = 44; *S. oralis* subsp. *dentisani*, n = 9; *S. oralis* subsp. *tigurensis*, n = 18; *S. pneumoniae*, n = 2; *S. mitis*, n = 5; *S. infantis*, n = 5; and *S. pseudopneumoniae*, n = 3) for which draft genome sequences have been published previously were obtained from the public database (*3*,*4*) and used as references. The species and subspecies of the reference strains were based on the results of the phylogenetic tree analysis of Velsko et al. (*4*). Annotation of the draft genomes was performed by using the DDBJ Fast Annotation and Submission Tool (*5*). A core gene alignment was generated by using Roary with the -e–mafft option and used for the determination of phylogenetic relationships. Phylogenetic trees were reconstructed with the maximum likelihood method by using IQ-TREE with 1,000 ultrafast bootstrap replicates (*6,7*). The trees were visualized by using iTOL version 3 (*8*). Sequences surrounding the capsule cluster were extracted from contigs of the draft genomes of ASP0312-Sp and SP2752. If necessary, PCR primer sets designed to cover gaps between the contigs and amplicon sequences were confirmed by using Sanger sequencing.

Homology Search of Pneumococcal Genes

Homology searches of 36 known pneumococcal virulence genes within the genomes of ASP0312-Sp and SP2752 were performed by using GENETYX (GENETYX, Tokyo, Japan). The

36 genes (encoded factors) were: *bgaA* (β-galactosidase), *cbp* (choline binding protein) *A*, *cbpC*, *cbpD*, *cbpE*, *cbpF*, *cbpG*, *cbpI*, *cbpJ*, *cbpL*, *cbpM*, *endoD* (endo-β-N-acetylglucosaminidase), *eno* (enolase), *fic* (Fic domain protein), *htrA* (high-temperature requirement protein A), *hyl* (hyaluronate lyase), *iga* (immunoglobulin A1 protease), *lyt* (autolysin) *A*, *lytB*, *nan* (neuraminidase) *A*, *nanB*, *pavA* (pneumococcal adhesion and virulence A), *pcpA* (pneumococcal choline binding protein A), *pfbA* (plasmin- and fibronectin-binding protein A), *pht* (polyhistidine triad protein) *A*, *phtB*, *phtD*, *phtE*, *ply* (pneumolysin), *psaA* (pneumococcal surface antigen A), *pspA* (pneumococcal surface protein A), *spxB* (pyruvate oxidase), *strH* (β-N-acetylglucosaminidase), *sub* (subtilase family protein), *zmpB* (zinc metalloproteinase B), and *zmpC* (zinc metalloproteinase C).

Nucleotide Sequence Accession Numbers

For ASP0312-Sp and SP2752, we deposited the whole-genome sequences (GenBank accession nos. DRX277946 and DRX232035, respectively), sequences around the capsule (GenBank accession nos. LC601604 and LC601606, respectively), and *hyl* sequences (GenBank accession nos. LC602142 and LC602144, respectively) in the DNA Data Bank of Japan (https://www.ddbj.nig.ac.jp).

References

1. Mundy LS, Janoff EN, Schwebke KE, Shanholtzer CJ, Willard KE. Ambiguity in the identification of *Streptococcus pneumoniae*. Optochin, bile solubility, quellung, and the AccuProbe DNA probe tests. Am J Clin Pathol. 1998;109:55–61. PubMed https://doi.org/10.1093/ajcp/109.1.55

2. Bankevich A, Nurk S, Antipov D, Gurevich AA, Dvorkin M, Kulikov AS, et al. SPAdes: a new genome assembly algorithm and its applications to single-cell sequencing. J Comput Biol. 2012;19:455–77. PubMed https://doi.org/10.1089/cmb.2012.0021

3. Kilian M, Tettelin H. Identification of virulence-associated properties by comparative genome analysis of *Streptococcus pneumoniae, S. pseudopneumoniae, S. mitis*, three *S. oralis* subspecies, and *S. infantis.* mBio. 2019;10:e01985-19. PubMed https://doi.org/10.1128/mBio.01985-19

4. Velsko IM, Perez MS, Richards VP. Resolving phylogenetic relationships for *Streptococcus mitis* and *Streptococcus oralis* through core- and pan-genome analyses. Genome Biol Evol. 2019;11:1077–87. PubMed https://doi.org/10.1093/gbe/evz049

5. Page AJ, Cummins CA, Hunt M, Wong VK, Reuter S, Holden MTG, et al. Roary: rapid large-scale prokaryote pan genome analysis. Bioinformatics. 2015;31:3691–3. PubMed https://doi.org/10.1093/bioinformatics/btv421

6. Edwards EK Jr, Edwards EK Sr. Ultraviolet radiation-induced suppression of contact hypersensitivity in relation to Padimate O and oxybenzone. J Invest Dermatol. 1989;93:708. PubMed https://doi.org/10.1111/1523-1747.ep12319927

7. Nguyen LT, Schmidt HA, von Haeseler A, Minh BQ. IQ-TREE: a fast and effective stochastic algorithm for estimating maximum-likelihood phylogenies. Mol Biol Evol. 2015;32:268–74. PubMed https://doi.org/10.1093/molbev/msu300

8. Letunic I, Bork P. Interactive tree of life (iTOL) v3: an online tool for the display and annotation of phylogenetic and other trees. Nucleic Acids Res. 2016;44(W1):W242–5. PubMed https://doi.org/10.1093/nar/gkw290

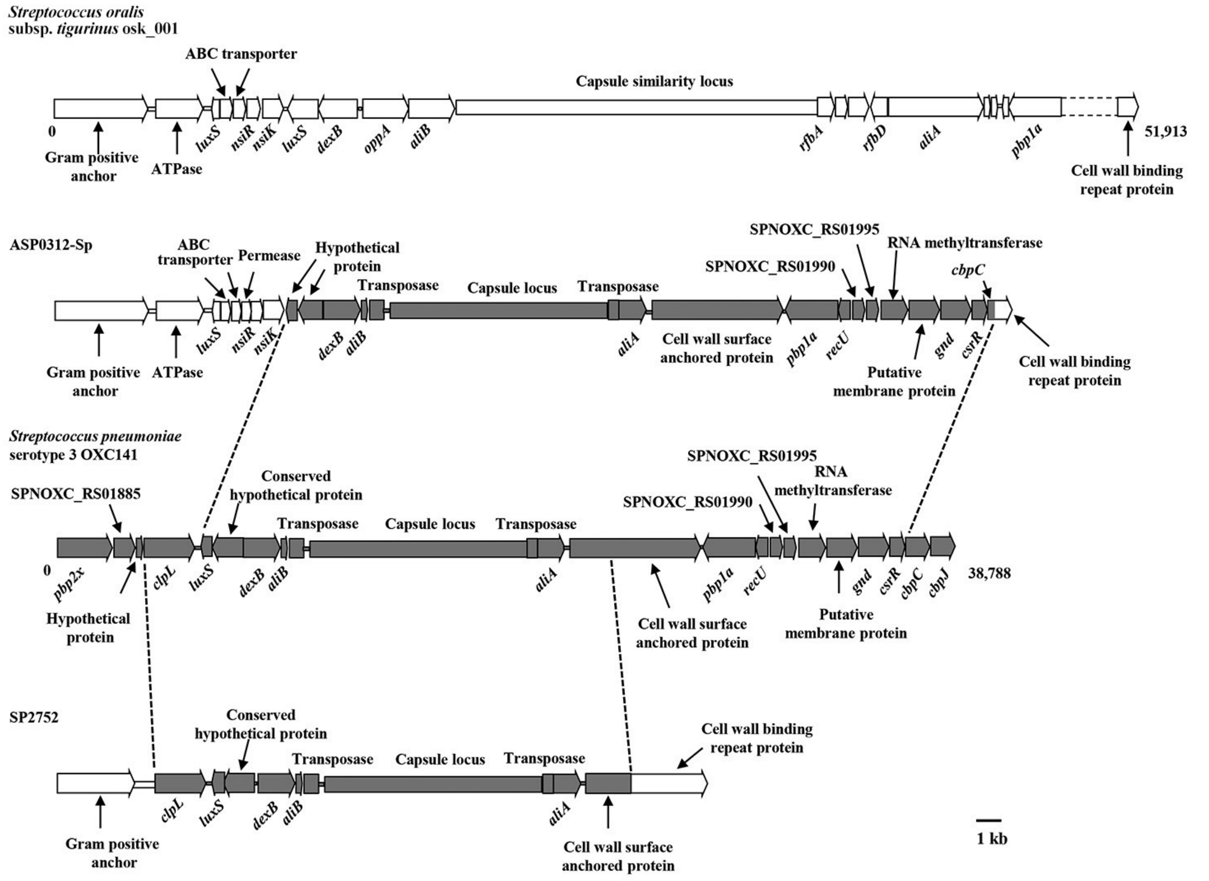

Appendix Figure. Linear genetic rearrangements of invasive *Streptococcus oralis* expressing serotype 3 pneumococcal capsule from 2 adult patients, Japan. Genetic rearrangements are shown between genes encoding the gram-positive anchor and cell wall binding repeat protein in strains ASP0312-Sp and SP2752. Gene clusters encoding the *Streptococcus pneumoniae* serotype 3 capsule are included in the region. Sequences at the corresponding locations in *S. oralis* subsp. *tigurinus* osk_001 (GenBank accession no.

AP018338.1) and *S. pneumoniae* serotype 3 OXC141 (GenBank accession no. FQ312027) were obtained from the National Center for Biotechnology Information database (https://www.ncbi.nlm.nih.gov) and used to determine the sites of recombination. All gene names used here are identical to those from the osk_001 and OXC141 strains. The white rectangles indicate the sequences of *S. oralis* subsp. *tigurinus* osk_001, and the gray rectangles indicate the sequences of *S. pneumoniae* serotype 3 OXC141. The recombination points are indicated by dashed lines. SPNOXC represents *Streptococcus pneumoniae* OXC41; the RS number after SPNOXC is the GenBank locus tag.
